# Is there an impact of feet position on squatting birth position? An innovative biomechanical pilot study

**DOI:** 10.1186/s12884-019-2408-2

**Published:** 2019-07-19

**Authors:** David Desseauve, Laetitia Fradet, Patrick Lacouture, Fabrice Pierre

**Affiliations:** 1 Department of Obstetrics and Gynecology and Reproductive Medicine, University Hospital, Poitiers University, CHU de Poitiers, 2 rue de la Milétrie, BP 577, 86021 Poitiers, France; 2 Pprime Institute - CNRS UPR 3346, Axis RoBioSS, Poitiers University, Poitiers, France; 3 Faculty of Biology and Medicine, Lausanne University, 1011 Lausanne, Switzerland; 4 Present Address: Department of Obstetrics and Gynecology, Centre Hospitalier Universitaire Vaudois (CHUV), 1011 Lausanne, Switzerland

**Keywords:** Squatting birth position, Feet posture, Biomechanics, Lumbar curve, Pelvic inlet plane, Motion capture system

## Abstract

**Background:**

The squatting birth position is widely used for “natural” birth or in countries where childbirth occurs in non-medical facilities. Squatting birth positions, like others, are roughly defined so a biomechanical assessment is required with the availability of noninvasive technology in pregnant women. In practice, we can observe spontaneously two kinds of squatting birth position: on tiptoes and with feet flat.

**Objective:**

To compare the impact of foot posture on biomechanical parameters considered essential in obstetrical biomechanics during a squatting birth position: on tiptoes versus with feet flat on the floor.

**Study design:**

Thirteen pregnant women beyond 32 weeks of gestational age who were not in labor were assessed during squatting birth position firstly spontaneously and secondly with the foot posture that was not taken spontaneously (on the tiptoes vs with feet flat). For each position, ANGle of flexion on the spine of the plane of the pelvis external conjugate **(ANGec),** hip flexion and abduction, and lumbar curve were assessed using an optoelectronic motion capture system and a biomechanical model adapted from the conventional gait model as well as a measuring system of the lumbar curve.

**Results:**

Spontaneously, 11 out of 13 women squatted on tiptoe at the first test. On tiptoes the hip flexion was lower than with feet flat (*p* < 0.02), whereas hip abduction was not significantly different (*p* = 0.28). A lower ***ANGec*** angle (*p* = 0.003) was noticed for the tiptoe position than feet flat. The lumbar curve (lordosis) was more marked for the squatting position on tiptoes than for the position with feet flat (*p* < 0.001). On tiptoes no woman had a pelvic inlet plane perpendicular to the spine and none had a flat back or kyphosis. No woman on tiptoes fulfilled the two conditions necessary for the position that we consider optimal.

**Conclusion:**

In squatting birth position, foot posture has a biomechanical impact on lumbar curve and pelvic orientation. When comparing squatting positions (on tiptoes vs feet flat), feet flat on the ground is closer to optimal birth conditions than on tiptoes.

## Background

At the beginning of the nineteenth century, Engelmann et al. observed that women not influenced by Western conventions mainly adopted the squatting birth position in the first and second phases of labor [1]. At present, as demonstrated by previous studies and in particular in European region, this position is rarely used in countries where birth medicalization is important. This position remains, however, widely used in countries where childbirth occurs in non-medical facilities (38.9% in Nepal in 2012) [2, 3].

According to Atwood, the squatting position is categorized among vertical positions [4]. It is well known that vertical positions have obstetrical benefits, particularly in terms of time to delivery with a reduction of obstetrical intervention. Among the hypotheses that might explain these results, we can envisage that the vertical position, like the squatting position, is closer to the theoretically best birthing position. This position enables the axis of progression to be perpendicular to the superior pelvic inlet plane and to encounter the fewest obstacles by flattening the dorsal hinge (or with kyphosis) as we have described previously [5]. To reach these optimal conditions, the pelvic inlet plane has to be close to perpendicular to the lumbar spine according to obstetrical theory [5].

From a biomechanical point of view, the squatting position is suffering of approximations about segmental positions (abduction/flexion of the thighs, lordosis) like other birth positions as we explained in a recent review [5]. Therefore, we can consider different kinds of squatting positions. The main difference that we can notice about them relates to the extension of the feet. We discerned two large families: squatting with the feet flat on the floor (this position was recently popularized under the name “Asian squat”), and squatting on tiptoe (sometimes called “western squat”) [6, 7].

**Fig. 1:**
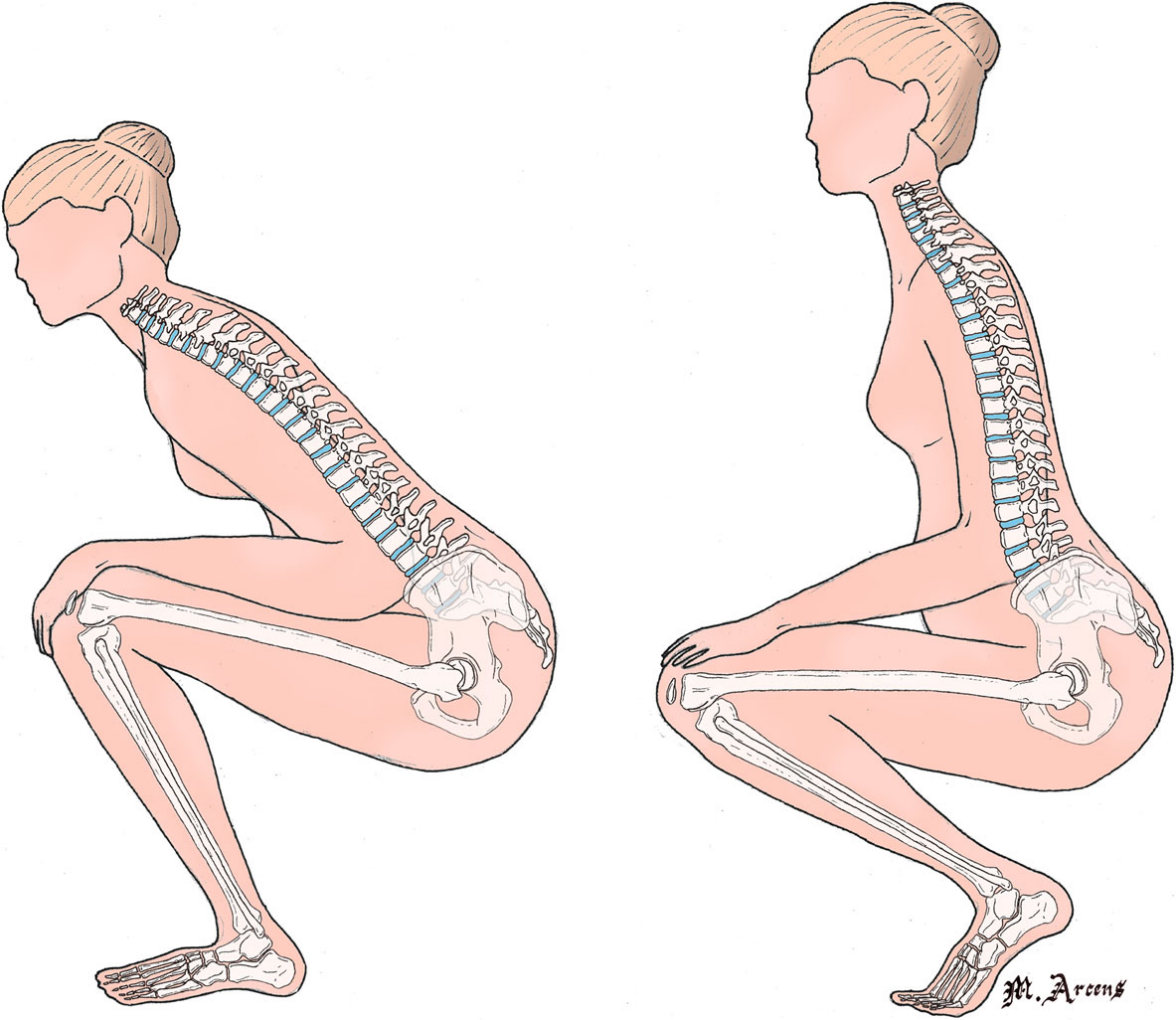
Examples of differences postures between two different squatting birth positions according to the flexion of the feet

These two kinds of foot position (illustrated by Fig. 1) are associated with different degrees of flexion and hip abduction, as highlighted by Hemmerich et al. [7]. However, in their study, these authors did not measure the consequences of these different attitudes of the feet on the position of the pelvis or spine.

We hypothesize that these two types of squatting positions could have different biomechanical consequences. Unfortunately, to our knowledge, no study on birthing positions has distinguished them and taken them into account in their results. Only Klein and Reid discussed the potential impact of the position of the feet on the squatting position, but did not conduct a study to characterize their impact on the “Pelvic Drive” described by Gold et al. in 1950 [8–10].

In this innovative biomechanical study, we compare the impact of two kinds of squatting position flexion according to the position of the feet (flat versus on tiptoe) on the biomechanical parameters (pelvic inlet plane and lordosis). This study answers the question: are all squatting positions equal in terms of obstetrical biomechanics?

## Methods

In this prospective comparative study, eligible participants were pregnant women older than 18 years and beyond 32 weeks of gestation, followed by physiological pregnancy consultation, with a body mass index under 40, and without inflammatory joint diseases or joint hypermobility syndrome, such as Marfan’s syndrome. In biomechanical studies, the number of subjects beyond 10 has an insignificant effect on statistical power [11]. Taking into account the risk of failure of data analysis or during experimentation, we approximated that 13 pregnant women were necessary in this study.

The study protocol was approved by the Ethics Committee of Poitiers Hospital (Comité de Protection des Personnes: 2013-1203-42) and by the French National Agency of Drug Safety (Agence Nationale de Sécurité du Médicament: B131-460-22). All women provided written informed consent.

**Fig. 2:**
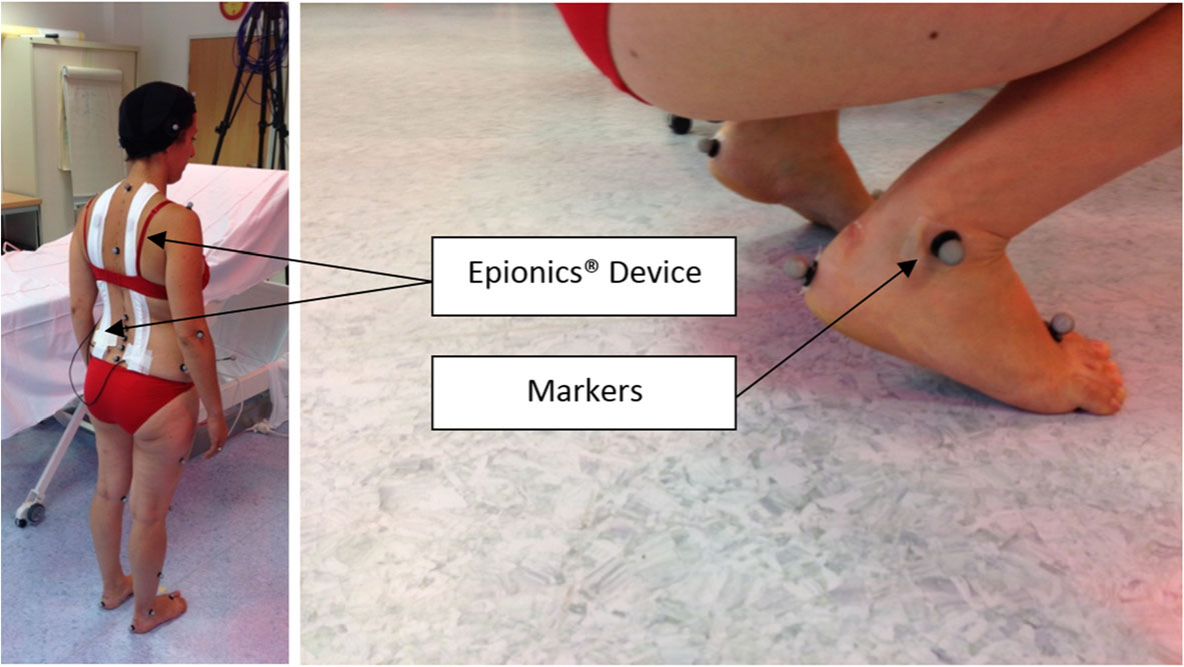
Example of setting markers

A full protocol description about this innovative methodology is available in a recent publication [12]. A traditional three-dimensional motion analysis was performed to analyze the position of the markers in space. It was based on an optoelectronic motion capture system consisting of 12 infrared cameras cadenced at 100 Hz (VICON, Oxford Metrics, UK). Thirty-three reflective markers were affixed using double-sided tape on anatomical landmarks according to an adapted version of the Helen Hayes’s marker set [12] (Fig. 2). To assess the position of the pelvis, we placed additional markers on the pelvis. An antenna fitted with three markers was positioned on the top of each iliac crest to provide a technical coordinate system, allowing the reconstruction of the pelvic markers if they were to be hidden during the experimentation. Marker trajectories were low-pass filtered using a double-pass Butterworth filter with a cutoff frequency of 10 Hz.

The lumbar curve was assessed by measuring the lordosis according to the Epionics SPINE system (Epionics Medical GmbH, Potsdam, Germany). This system consists of two flexible sensor strips that use strain gauge sensors located alongside flexible circuit board strips. The positioning of the system is standardized. According to this measure, a lordosis of 0° corresponds to a back perfectly flattened. The data acquisition (50 Hz) was transmitted in real time via Bluetooth to a local personal computer [12]. This biomechanical study took place in an experimental setting (i.e., not during labor).

In practice, women were asked to perform two types of squatting positions. We first asked the subjects to spontaneously squat without further instruction and to stay in this position. Data acquisition began when the subject was stabilized for at least 3 sec. Then we asked the subject to stand up to take the squatting position that was not spontaneously adopted at the time of the first acquisition. For all subjects, we had an acquisition of two different squatting positions (feet flat and on tiptoes). For the squatting position carried out with feet flat, which was often more difficult to maintain, we offered the women a stick to stabilize themselves.

**Fig. 3:**
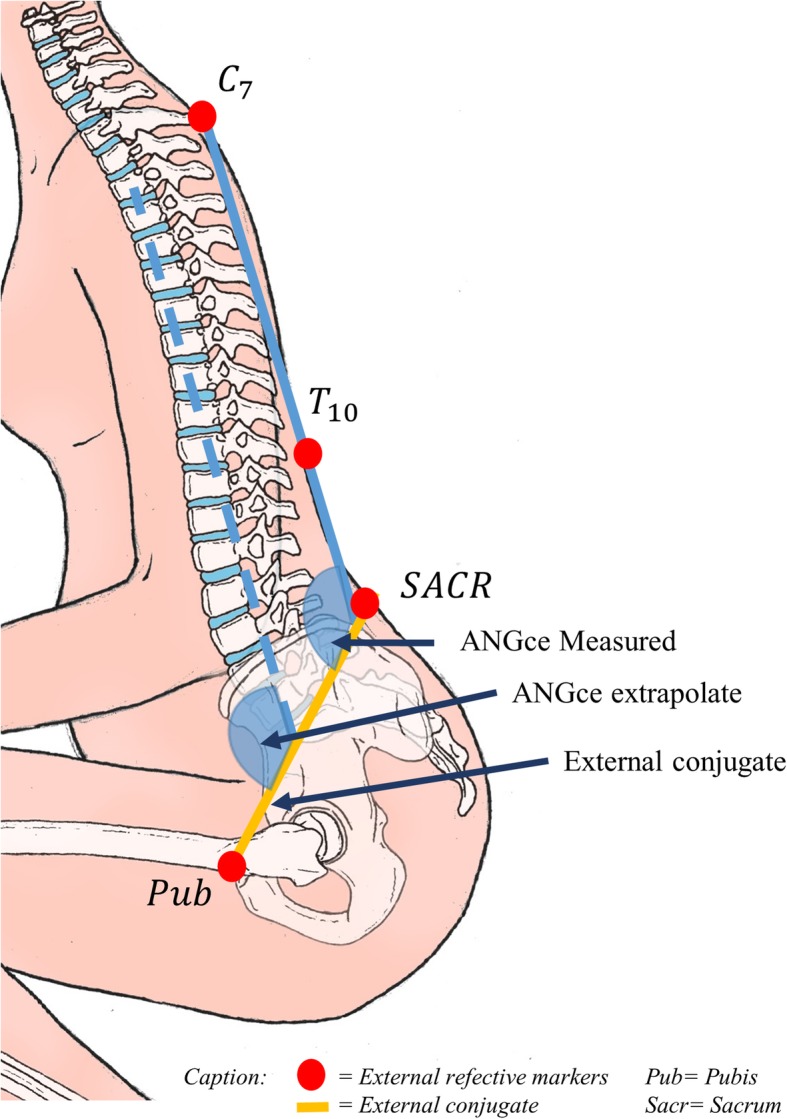
Definition of ANGec and external conjugate

A custom Matlab code (MathWorks Inc., Natick, MA) was used to merge data from Epionics and VICON systems and to extract the required data. We defined a plane following the external conjugate diameter using the two markers placed on the posterosuperior iliac spines and the marker placed on the superior edge of the pubic symphysis. The hip joints angles (flexion and abduction) were obtained as defined by the conventional gait model. The flexion of the plane of the external conjugate on the spine (***ANGec***) was defined in the sagittal plane as the angle between the external conjugate and the line defined by the markers placed on the 7th cervical and the 10th thoracic vertebrae (Fig. 3). ***ANGec*** have by convention negative value until the pelvic inlet plane was perpendicular to the lumbar spine. Beyond ***ANGec*** were positive.

The lumbar curvature was measured during the two different positions for each subject.

For each woman, conditions for optimal birth as defined above were assessed. As a reminder, we considered that the back was flat from − 3 ° of lumbar lordosis or in kyphosis when values were positive. The plane of the pelvic inlet plane is perpendicular to the rachis when ***ANGec*** reach +/− 5 ° (taking into account the precision of the measurements).

All values obtained for the two types of squatting position (feet flat vs on tiptoes) were compared using a Wilcoxon matched-pairs signed-ranks test. The significance level was defined as *p* < 0.05.

## Results

As none of the 13 participants withdrew after giving informed consent, they have all been assessed. The mean age of the participants was 32.8 (Standard Deviation (SD) 2.8) years, and the mean of the term at the inclusion was 34.0 (SD 0.7) weeks of amenorrhea. The mean body mass index was 26.0 (SD 0.8) kg.m^− 2^. Seven participants (60%) were primiparous.

Spontaneously, 11 out of 13 women squatted on tiptoe at the first test. All the participants were able to squat whatever the initial posture (passing from tiptoe to feet flat and vice versa for two women).

According to Table 1, segmental posture (hip, pelvis) were very different between the two types of squatting position. On tiptoes hip flexion was lower than with feet flat (*p* = 0.02), whereas hip abduction was not significantly different (*p* = 0.28).

**Table 1:** Average values of the parameters for the pelvis, lumbar spine, and thighs, according to the two squatting birth positions tested in the study (mean, SD)

	Squatting birth position		*p value*
	On tiptoes $\overline{X}$ [SD]	Flat feet $\overline{X}$ [SD]	
Hip flexion (°)	103 [15]	125 [15]	0.02
Hip Abduction (°)	29 [11]	28 [10]	0.28
ANGec (°)	− 49 [13]	− 34 [9]	0.003
Lumbar curve (°)	−18 [14]	− 1 [12]	< 0.001

A lower ***ANGec*** angle (*p* = 0.003) was noticed for the position on tiptoes than feet flat.

There was also a difference in the lumbar curve (lordosis), which was higher for the squatting position on tiptoes than for the position with feet flat (*p* < 0.001).

On tiptoes no woman had a pelvic inlet plane perpendicular to the spine (***ANGec*** = 0°+/− 5°) and none had a flat back or kyphosis (lumbar curvature = 0 +/− 3 °). No woman on tiptoes fulfilled the two conditions necessary for the position that we consider optimal (Table 2).

**Table 2:**
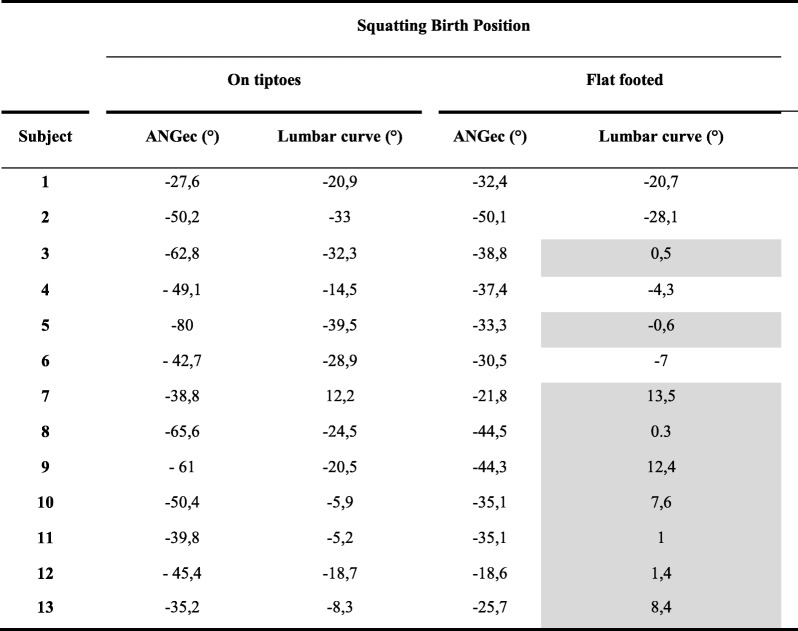
Values of the flexion of the plane of the external conjugate on the spine (***ANGec***), and lumbar curve for each subjects according to the two squatting birth positions assessed

The gray values correspond to the values of optimal lumbar curvature or ANGec

The feet flat squatting position enabled an angle of the pelvic inlet plane significantly ***(ANGec)*** closer to the perpendicular than the squatting tiptoe position.

With the feet flat, no woman had the pelvic inlet plane perpendicular to the spine (***ANGec*** = 0°+/− 5°). Regarding lumbar curve, 9 out of 13 women had flat backs (subject 3, 5, 8, 10 to 12) or kyphosis (subject 7, 9, 13) (Table 2).

## Discussion

To our knowledge, this study is the first to demonstrate the impact of foot position on global posture during a squatting birth position. This impact concerned essentially pelvic orientation and lumbar curve, which are considered as pivotal parameters to reach optimal birth position [5].

By definition, as opposed to the standing position, the squatting position referred to a posture in which the knees are flexed. This flexion resulted in a remoteness of the legs and thighs relative to the vertical. This singular mobilization of the lower limbs lead to an inclination of the trunk forward, made possible by displacing the center of gravity in the support polygon. By limiting muscle activation of the quadriceps muscles, the glutes can lean on the calves, making this posture as comfortable as possible. When the feet are flat, and the glutes rest on calves, the heel cord and gastrocnemius muscles are particularly stretched, the legs (shins) are more vertical. As a result, additional adjustments may occur, such as increasing hip flexions. This additional hip flexion, and the trunk adjustments necessary to maintain balance, had implications for pelvic position and lumbar curve. Our results showed that these adaptations resulted in a pelvic inlet plane closer to perpendicular and a correction of lumbar lordosis. These two conditions were considered as necessary to reach an “optimal” position.

To the question “were all squatting positions equal?” we answered no. We highlighted the importance of controlling the position of the feet in order to make the squatting birth position as favorable as possible biomechanically. In another field of study, it has been shown that the squatting position, in particular in hyperflexion, was the most favorable to obtain a recto-anal canal close to rectitude during defecation. In this position lower abdominal pressure is necessary to defecate [13]. Without direct comparison, we can hypothesize that in this position there was a lower resistance to the fetal progression. The optimal lumbar and pelvic birth conditions were not reached by subjects in this study. But, squatting with feet flat led to approaching them in particular for lumbar curve. The plane of the pelvic inlet plan was closer to be perpendicular to the lumbar spine in the squatting birth position with the feet flat. The squatting position with feet flat “naturally” approached the optimal conditions.

Among our population, three women in the squatting position with feet flat had beyond a flat back, a kyphosis (a round back). This posture is not problematic from a biomechanical point of view, because it did optimize the “obstetric chute” that we defined in a previous review [5]. Moreover, increasing kyphosis didn’t close the angle between the pelvic inlet plane and the spine. We can hypothesize that kyphosis would be necessary to reach the optimal birth position.

In this study, almost all of our subjects did not spontaneously adopt the squatting position with feet flat. The women’s natural choice of position was not the best biomechanically. Global position is nowadays an utter nonsense. All segmental positions must be defined and used to better assist women’s position choice. A better definition, “measuring” position is a necessity in all future works on birth position. A better birth definition will lead to a better assessment of the impact of position on obstetrical outcomes.

A major limit is that this pilot study focused on women at the third trimester near term, but not in labor. The next step in our research should be to confirm our results during labor. In the future, the final step should be to confirm the impact of feet position during the squatting birth position correlated to an assessment of obstetrical outcomes (labor duration, caesarean section, duration of the second phase of labor).

Our innovative biomechanical approach could have a real impact on supporting birth in alternative positions. Squatting birth position is one of the most common positions in countries with low medicalization [3]. Simple advice about feet posture could have impact on obstetrical outcomes, particularly when an obstructed labor occurs. This kind of research must be supported because avoiding C-section, using simple posture advice, in countries with poor healthcare accessibility or where C-section is associated with a high risk of maternal morbidity or mortality, should be a smart obstetrical approach.

In well-resourced countries the empowerment of the birthing experience for women who wish to give birth as naturally as possible and in security resulted in the introduction of the “natural birth space” in the classic labor ward, or birth center in maternity. In these units, women have the choice to give birth in the position that they wish. Supporting women in their choice and advising on an optimal position according to our research should be a new challenge for future birth-care providers.

## Conclusion

In squatting birth position, foot posture had a biomechanical impact on lumbar curve and on pelvic orientation. When comparing squatting positions (on tiptoes vs feet flat), feet flat is closer to the optimal birth condition than on tiptoes. The clinical impact of these new considerations has to be explored in future researches.

## Data Availability

The datasets used during the current study are available from the corresponding author on reasonable request.

## Abbreviations

ANGec: ANGle of flexion on the spine of the plane of the pelvis external conjugate
SD: Standard Deviation
